# Bidirectional crosstalk between the nervous system and the tumour microenvironment: mechanisms, feedback loops and therapeutic opportunities

**DOI:** 10.3389/fcell.2026.1895424

**Published:** 2026-08-04

**Authors:** Guicheng Kuang, Zhenghaonan Qiu, Lingxiao Li, Hang Ji, Yi Liu

**Affiliations:** 1 Department of Neurosurgery, West China Hospital, Sichuan University, Chengdu, Sichuan, China; 2 West China School of Medicine, Sichuan University, Chengdu, Sichuan, China

**Keywords:** cancer neuroscience, neuro-immune axis, neuroplasticity, neurotransmitters, therapeutic strategies, tumour microenvironment

## Abstract

The nervous system is increasingly recognized as an active and integral component of the tumour microenvironment (TME), rather than a passive bystander affected by tumour invasion. Emerging evidence indicates that neural inputs shape tumour behaviour through both direct and indirect mechanisms. Neurotransmitters, neuropeptides and neurotrophic factors act on tumour cells, stromal cells, endothelial cells and immune cells to regulate proliferation, invasion, metastasis, angiogenesis, metabolic reprogramming and immune evasion. In parallel, the TME feeds back to the nervous system through inflammatory mediators, extracellular vesicles, axon guidance molecules and metabolic signals, thereby driving axonogenesis, tumour innervation, Schwann cell reprogramming, neuronal hyperexcitability and synaptic remodelling. These reciprocal interactions establish dynamic neuro–immune–metabolic feedback loops that sustain tumour progression and therapeutic resistance. Particularly in glioma and other highly innervated malignancies, activity-dependent neuron–tumour communication further highlights the functional integration between neural circuits and cancer. In this Review, we summarize the structural and molecular basis of neural components within the TME, discuss neurotransmitter receptor-mediated signalling and indirect regulation of immune, vascular, stromal and metabolic niches, and outline how tumour-derived signals remodel peripheral and central neural systems. We further highlight emerging therapeutic opportunities targeting β-adrenergic signalling, neurotrophin pathways, extracellular vesicle-mediated tumour innervation, Schwann cell-associated perineural invasion circuits, and neuron–tumour synaptic coupling. Finally, we discuss current translational challenges, including tumour-type heterogeneity, context-dependent neural effects, evidence-level heterogeneity and the need for spatially resolved biomarkers, and propose that incorporating the neural dimension into future mechanism-guided studies may inform biomarker-stratified trials and symptom-oriented interventions, with the long-term goal of improving both tumour control and neurological outcomes.

## 1 Introduction

The historical recognition of tumour–nerve interactions predates the modern field of cancer neuroscience and can be traced back to nineteenth-century pathological and histological studies (Zhang et al., 2022; Maroldi et al., 1999). In 1862 Neumann reported secondary cancroid infiltration of the mental nerve in lower-lip carcinoma, thereby providing early anatomical evidence for tumour spread along peripheral nerves (Neumann, 1862). By the late nineteenth century, Hugh H. Young employed a modification of Ehrlich’s methylene-blue vital staining method to reveal nerve fibres within tumour tissue, extending these observations from perineural spread to the histological recognition of tumour innervation (Young, 1897). These early accounts were primarily descriptive and regarded nerves largely as anatomical structures affected by tumour extension or simply embedded within tumour tissue. Only much later did experimental oncology begin to reinterpret tumour-associated nerves as active, plastic and functionally relevant components of the tumour microenvironment.

Over the following decades, nerves were gradually recognized as an important component of the tumour microenvironment, and the phenomenon of perineural invasion (PNI) was identified, confirming that tumour-secreted factors can induce peripheral neuritis in regions not directly infiltrated by the tumour (Li et al., 2021). At the same time, neurotrophic factors such as nerve growth factor (NGF) and brain-derived neurotrophic factor (BDNF) were found to promote tumour proliferation, migration, and invasion (Aloe et al., 2016; Micera et al., 2007). However, these observations did not fundamentally change the conceptual framework of nerve-tumour interactions until experimental studies demonstrated that tumours can promote infiltration by parasympathetic cholinergic fibres and that the densities of sympathetic and parasympathetic fibres in both the tumour microenvironment and the surrounding normal tissues are each associated with clinical outcomes (Magnon et al., 2013). These findings were subsequently validated in models of prostate cancer (March et al., 2020), breast cancer (Liu et al., 2024; Zarrer et al., 2020), gastric cancer, and lung cancer. On the other hand, sympathetic activation engages adrenergic neural signalling, which is required during the early stages of tumour progression and for the initiation of the angiogenic switch (March et al., 2020), whereas parasympathetic activation engages cholinergic neural signalling, which may accelerate tumour dissemination and metastasis (Ni et al., 2023).

With continued advances in this field, the pivotal role of the nervous system in tumour initiation, progression, and metastasis has become increasingly apparent. This field has emerged from a deeper understanding of the dynamic regulation of the tumour microenvironment (TME), emphasizing that the nervous system is no longer viewed as a passive victim of cancer, but rather as a component of a bidirectional regulatory network with tumour cells that promotes invasion, metastasis, and therapeutic resistance (Wang et al., 2022). Tumour cells, immune cells, vascular endothelial cells, stromal cells, and other constituents of the TME are all influenced by neural signals; conversely, tumour cells can remodel the neural landscape by releasing neurotrophic factors or reshaping synaptic architecture, thereby inducing neuronal hyperexcitability and cognitive dysfunction (Venkatesh et al., 2019). For example, in colorectal cancer, stress signalling mediated by the beta2-adrenergic receptor (beta2-AR) activates downstream PI3K/AKT and MAPK pathways, promoting tumour-cell proliferation and angiogenesis while suppressing antitumour immunity (Varghese et al., 2025). Similarly, in hepatocellular carcinoma, the sympathetic nervous system accelerates tumour progression through norepinephrine (NE) release, thereby regulating lipid metabolism and immunosuppression (Tang et al., 2025).

The emergence of this bidirectional regulatory network has been driven not only by breakthroughs in basic research but also by support from epidemiological evidence.

Tumours with a high nerve density often have a worse prognosis; for example, in patients with breast cancer and prostate cancer, tumoural neural infiltration is associated with increased metastatic risk and reduced survival (Chuang et al., 2019). Clinical and histopathological evidence also supports the prognostic relevance of tumour-associated nerves in selected cancer types. In breast cancer, intratumoural nerve fibres have been associated with NGF production and lymph-node invasion, suggesting a relationship with increased metastatic potential (Pundavela et al., 2015). In prostate cancer, increased autonomic nerve density in and around tumour tissue has been linked to more aggressive disease and poorer clinical outcome (Magnon et al., 2013; Olar et al., 2014). Glioma is frequently accompanied by seizures, which may be related to neurogliomal synapses (NGSs), in which synaptic signalling activates the PI3K/mTOR pathway through glutamate and promotes tumour proliferation (Wang Y. et al., 2025). Moreover, neuronal activity-induced synaptic integration has been proposed as a core mechanism underlying glioma progression, underscoring the promise of cancer neuroscience as an emerging field (Monje et al., 2020). More recent studies have further revealed neuro-immune cross-talk, whereby neurotransmitters regulate immune-cell polarization to form an immunosuppressive microenvironment and thereby provide new targets for tumour immune evasion (Amit et al., 2025). Collectively, these findings indicate that bidirectional regulatory mechanisms influence key processes such as metabolic reprogramming, angiogenesis, and immune escape, opening new avenues for precision therapy and providing complementary strategies to overcome the limitations of conventional treatment. For example, GABA oxidation and succinic semialdehyde dehydrogenase (SSADH) activity may represent additional therapeutic targets in glioblastoma (Hujber et al., 2018). Given that glutamatergic neuron–glioma synapses provide AMPA receptor-dependent excitatory input to glioma cells, AMPA receptor antagonism has emerged as a mechanism-based strategy to weaken neuron–tumour synaptic coupling; the PerSurge/NOA-30 phase II trial is evaluating perioperative perampanel treatment in patients with progressive glioblastoma (Venkataramani et al., 2019; Heuer et al., 2024).

In models of liver cancer, sympathetic blockade decreases fatty-acid oxidation and enhances chemosensitivity (Huang et al., 2022). Nevertheless, an important challenge lies in the systemic impact of neural interventions, as broad blockade may produce cognitive adverse effects. Future studies should therefore focus on subtype-specific targeting, such as the development of selective beta-AR antagonists or the use of nanodelivery systems to precisely modulate TME-associated neurons (Li W. et al., 2025) In addition, integrating multi-omics data, such as spatial transcriptomics, may help identify biomarkers of nerve-tumour interactions and guide individualized therapy. Overall, the field is driving a shift from a tumour-centred view to a systems-network perspective. This transition not only deepens our understanding of tumour heterogeneity but also provides a foundation for the development of neuromodulatory therapies. By targeting nerve-tumour interactions, we may be able to overcome the limitations of conventional treatment and improve patient outcomes.

Despite considerable progress in the field, the core purpose of this Review is to organize the discussion around the following four aspects: innervation-driven tumour progression, which explains how sympathetic, parasympathetic and sensory neural signals directly or indirectly promote tumour proliferation, angiogenesis, immune suppression, stromal remodelling and metabolic reprogramming; tumour-induced neuralization, whereby tumour and stromal cells can remodel local neural structures through secreting neurotrophic factors, extracellular vesicles and axon-guidance molecules, and by reprogramming Schwann cells, thereby exacerbating tumour innervation and perineural invasion; synaptic and electrical coupling, which summarizes the activity-dependent functional connection between neurons and malignant cells-a feature particularly prominent in glioma and brain metastases, where neuron–tumour synapses, tumour microtubes and activity-regulated secreted factors support tumour growth while altering neuronal excitability; and systemic neuroimmune feedback, in which tumour-derived inflammatory, metabolic and sensory signals activate peripheral afferents, central neural circuits, the hypothalamic–pituitary–adrenal axis and autonomic efferent pathways, subsequently feeding back to reshape antitumour immunity, clinical symptoms and therapeutic responses. This loop-based analytical framework constitutes the core organizing thesis of the present Review and distinguishes it from previous reviews in the field by simultaneously integrating local tumour neuro-microenvironmental regulatory mechanisms with systemic cross-organ communication pathways.

## 2 Neural regulation of the tumour microenvironment

### 2.1 Structural basis of neural components in the tumour microenvironment

The nervous system is not only a central regulatory network for information integration and stress responses in the body, but is also increasingly recognized as an important component of the tumour microenvironment (TME), participating in tumour initiation and progression, immune regulation, and the shaping of therapeutic responses through structural remodelling and signal transmission (Zahalka and Frenette, 2020; Boilly et al., 2017). Anatomically and functionally, the nervous system can be divided into the central nervous system (CNS) and the peripheral nervous system (PNS). The PNS includes somatic sensory and motor nerves as well as the autonomic nervous system (ANS), which is composed of the sympathetic nervous system (SNS) and the parasympathetic nervous system (PSNS; of which the vagus nerve is a major component); the enteric nervous system (ENS), which has a relatively independent regulatory capacity, is also present (Zahalka and Frenette, 2020; Vaes et al., 2022). Within the TME, these neural branches, together with their associated Schwann cells, nerve sheath structures, and neuro-immune interfaces, constitute ‘tumour-associated neural components’. Through axonal entry into or around tumours, release of neurotransmitters or neuropeptides, and multidirectional interactions with tumour cells, vascular endothelial cells, fibroblasts, and immune cells, they form a regulatory axis linking nerves, tumours, and stromal/immune elements (Zahalka and Frenette, 2020; Boilly et al., 2017).

Structurally, neo-innervation and axonogenesis in tumour tissue, and perineural invasion (PNI), are two common yet biologically distinct phenomena: the former emphasizes tumour-induced entry of nerve fibres into the tumour microenvironment, thereby increasing intratumoural nerve density, whereas the latter refers to the migration and extension of tumour cells along the perineurium or perineural spaces. These processes may reinforce one another and are associated with metastatic risk and recurrence (Boilly et al., 2017). In the prostate, for example, remodelling and infiltration of autonomic nerves contribute to tumour progression, suggesting the existence of a “nerve-dependent tumour niche” (Magnon et al., 2013). Subsequent studies further showed that norepinephrine released from sympathetic nerves can trigger the angiogenic switch through beta-adrenergic signalling in endothelial cells, altering vascular metabolism within the TME and promoting endothelial metabolic reprogramming, tumour growth, and thus underscoring the importance of neuro-endothelial metabolic interactions in the TME (Zahalka et al., 2017). In gastrointestinal tumours, nerve-tumour interactions are also prominent. In gastric cancer, tumour stage correlates with nerve density, and vagal innervation promotes gastric tumourigenesis through Wnt signalling mediated by the M3 receptor in stem cells; vagotomy reduces the risk of gastric cancer, suggesting that neural input exerts a permissive effect on certain epithelial tumours (Zhao et al., 2014). In colorectal cancer (CRC), neural components within the TME also play an important role in disease progression. PNI and increased tumour nerve density are associated with poor outcomes, and denervation suppresses tumour growth in animal models, suggesting that chemical denervation can effectively inhibit rectal tumour growth *in vivo* (Wang H. et al., 2025). Mechanistically, on the one hand, norepinephrine induces ADRB2 (beta2-adrenergic receptor)-dependent NGF secretion by tumour cells, cancer-associated fibroblasts, and other stromal cells in the TME, thereby increasing sympathetic innervation and norepinephrine accumulation within tumours and establishing a vicious cycle. On the other hand, epinephrine directly accelerates CRC growth via ADRA2A/Gi-mediated activation of Yes-associated protein (YAP). Together, these observations provide direct evidence for a “nerve-stroma-tumour” feedback loop and suggest a potential mechanistic basis for targeting this axis in rectal cancer therapy (Kobayashi et al., 2025).

From a functional perspective, neurotransmitter and neuropeptide signalling constitutes a key pathway through which the PNS regulates the TME. For example, norepinephrine released by the SNS acts through beta-adrenergic receptors not only to influence the states of tumour and stromal cells, promoting angiogenesis and metabolic adaptation (Zahalka et al., 2017), but also to alter the metabolism of myeloid-derived suppressor cells (MDSCs) through beta2-adrenergic receptor signalling and enhance their immunosuppressive activity, thereby weakening antitumour immunity (Mohammadpour et al., 2021). In addition, the accumulation and acidification of tumour metabolites such as lactate can suppress CD8^+^ T-cell and NK-cell effector functions while supporting immunosuppressive cell populations; therefore, when sympathetic signalling drives the vascular-metabolic niche towards a highly glycolytic, high-lactate state, it often heralds immunosuppression within the TME (Zahalka et al., 2017; Gu et al., 2025). The PSNS and vagal cholinergic pathways display marked organ- and disease-stage-dependent duality. On the one hand, acetylcholine (ACh) can act on tumour cells through muscarinic acetylcholine receptors (mAChRs) and exhibits tumour-promoting proliferative signalling in gastric cancer models (Zhao et al., 2014); on the other hand, in pancreatic ductal adenocarcinoma (PDAC), enhanced cholinergic-CHRM1 signalling suppresses tumorigenesis, reduces cancer stem cell-like traits, and remodels myeloid inflammation, thereby inhibiting tumour development through MAPK and PI3K/AKT signalling (Renz et al., 2018) and directly as well as indirectly suppressing PDAC-cell growth. Thus, therapies that stimulate muscarinic receptors may be beneficial in PDAC. Neuropeptides released by sensory nerves are likewise crucial in immune regulation. For example, interactions between melanoma cells and nociceptive neurons enhance neurite outgrowth, responsiveness to noxious ligands, and neuropeptide release; CGRP released by tumour-associated nociceptors promotes CD8^+^ T-cell exhaustion and impairs immune surveillance through its receptor axis, whereas blockade of this pathway can enhance antitumour immunity (Balood et al., 2022).

Notably, communication between nerves and tumours is not limited to paracrine neurotransmission. In CNS tumours, more direct electrophysiological coupling has been observed: glioma cells can form AMPA receptor-dependent excitatory synapse-like connections with neurons and become integrated into neural circuits, through which neuronal activity drives tumour growth and invasion by electrophysiological signalling; activity-dependent secreted factors further amplify this positive feedback loop (Venkatesh et al., 2019; Venkatesh et al., 2015). The direct and indirect mechanisms by which the nervous system shapes the tumour microenvironment are discussed in greater detail below.

### 2.2 Direct regulation mediated by neurotransmitters and their receptors

**FIGURE 1:**
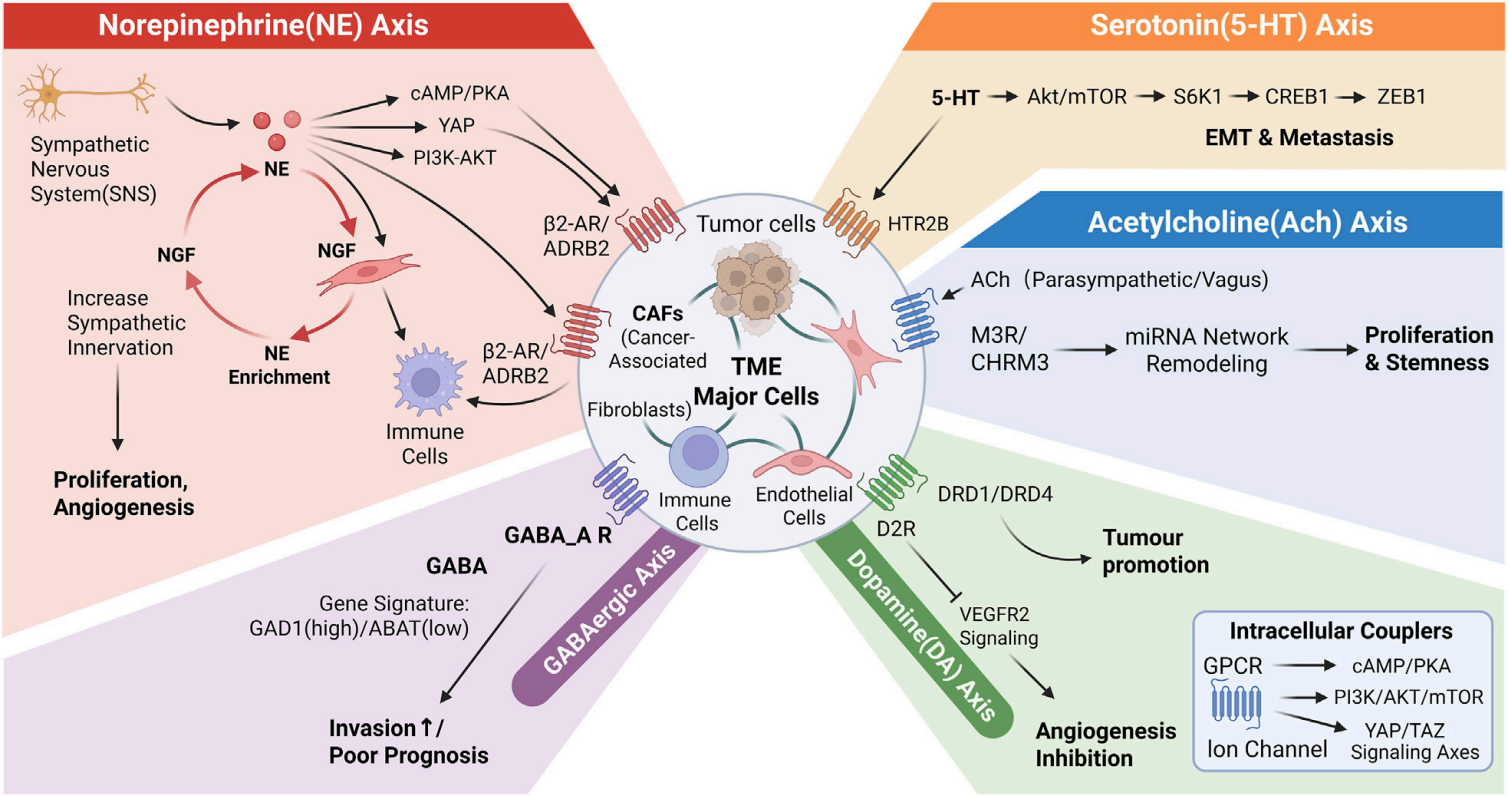
Neurotransmitter and Receptor-Mediated Direct Communication Network Schematic illustration of five neurotransmitter axes (norepinephrine, serotonin, acetylcholine, GABA, dopamine) mediating neural modulation of tumour microenvironment constituent cells: tumour cells, cancer-associated fibroblasts, immune cells and endothelial cells. Each axis features defined ligand-receptor pairs with unidirectional signal transduction to regulate proliferation, metastatic capacity, invasiveness and angiogenesis.

Direct neurotransmitter-receptor communication is one of the central mechanisms by which the nervous system shapes the tumour microenvironment (TME). Multiple neurotransmitters, including catecholamines, GABA, acetylcholine, serotonin, and sensory neuropeptides, together with their receptors, are widely expressed on tumour cells, cancer-associated fibroblasts (CAFs), endothelial cells, and immune cells. Most of these receptors belong to the G-protein-coupled receptor (GPCR) family or to ligand-gated ion channels, and they can rapidly trigger signalling axes such as cAMP/PKA, PI3K/AKT/mTOR, and YAP/TAZ, thereby exerting major effects on cell proliferation, epithelial-mesenchymal transition (EMT), angiogenesis, metabolic reprogramming, and immunosuppression (Kobayashi et al., 2025; Komine et al., 2025; Cole and Sood, 2012) (Figure 1).

Within catecholaminergic signalling, norepinephrine (NE) derived from sympathetic nerves drives coordinated pro-tumour effects across multiple cellular compartments through beta-adrenergic receptors. As discussed above, the ‘nerve-stroma’ mechanism in CRC is illustrative: NE induces ADRB2-dependent NGF secretion by CAFs, thereby enhancing sympathetic innervation within tumours and leading to local NE accumulation. At the same time, catecholamine stimulation accelerates CRC growth via ADRA2A/Gi-mediated YAP activation, whereas CAF-derived NGF directly promotes tumour-cell proliferation through Trk-PI3K/AKT signalling. In mouse models, Trk inhibitors downregulate YAP/AKT activity and suppress CRC progression (Kobayashi et al., 2025). At the neurotransmitter-receptor level, this mechanism integrates neural fibre recruitment, stromal remodelling, and tumour-intrinsic signalling into a unified circuit and represents an important exploration of potential drug targets for neuro-targeted combination therapy.

Consistent with experimental evidence, clinicopathological studies also support beta2-AR as a stratification marker in CRC. In a cohort of patients with advanced or recurrent CRC, high tumoural beta2-AR expression was associated with shorter progression-free survival and with reduced sensitivity to oxaliplatin-containing or anti-VEGF-based regimens, whereas combined propranolol and bevacizumab treatment significantly reduced tumour size in CRC xenograft models (Komine et al., 2025). Given that beta-adrenergic signalling can diminish cytotoxic immunity and promote an immunosuppressive microenvironment in multiple tumour types through effects on immune and stromal cells (Cole and Sood, 2012), future strategies combining beta-blockade with antiangiogenic therapy or immunotherapy will require precise patient selection based on intratumoural receptor-expression profiles and innervation characteristics. Repurposing existing beta-blockers may therefore be beneficial for tumours that are resistant to current treatment regimens.

Beyond catecholamines, GABAergic signalling has received increasing attention in oncology in recent years. In tumour organoid-on-chip systems and functional assays, GAD1 knockdown or pharmacological inhibition reduced invasiveness, whereas GABA increased the proliferation rate of glioma cells (Strelez et al., 2025). Notably, the directionality of GABA effects on tumour-cell proliferation and migration is not uniform: *in vitro* experiments show that GABA agonists can inhibit CRC-cell proliferation, yet high GABA levels *in vivo* often promote tumour growth (Tang et al., 2025). This suggests that GABA-dependent effects are likely co-determined by tumour genetic background, receptor subtype, and multicellular interactions, and that GABA oxidation may represent an additional therapeutic target in cancer.

Among other neurotransmitters, serotonin (5-HT) and the 5-HT receptor HTR2B also play important roles in tumours. High HTR2B expression in CRC tissues is closely associated with poor prognosis; mechanistically, HTR2B promotes CREB1 phosphorylation and transcriptional activation of ZEB1 through the Akt/mTOR-S6K1 pathway, thereby enhancing the invasive and metastatic capabilities of tumour cells and promoting EMT, whereas HTR2B antagonists suppress metastasis and EMT in mouse metastasis models (Li et al., 2024). In addition, further evidence suggests that HTR2B marks CRC-cell populations with enhanced proliferative capacity, invasive potential, and EMT status, and that its functional output is strongly influenced by the extracellular matrix (ECM) state (Carmi et al., 2025), highlighting the spatial heterogeneity and interdependence of the neurotransmitter-receptor axis.

Acetylcholine (ACh) signalling likewise participates in biological shaping of the tumour microenvironment. In colon cancer, aberrant muscarinic receptor expression is closely associated with pro-tumour behaviour. For example, activation of the M 3 muscarinic receptor (M3R) by ACh reshapes tumour-associated miRNA networks, stimulates miR-222 expression and colon-cancer-cell proliferation through PKC/p 38 MAPK signalling, and can be blocked by atropine, indicating that cholinergic signalling contributes to tumour progression through post-transcriptional regulation (Larabee et al., 2022).

From the classical dopamine perspective, dopamine can induce VEGFR2 internalization through the D2 receptor, thereby inhibiting VEGF-driven vascular permeability and angiogenic signalling (Basu et al., 2001). However, the role of dopamine in the tumour microenvironment appears complex, dose dependent, and bidirectional (Sarkar et al., 2022; Peters et al., 2014). Tumour cells commonly express DRD1-DRD 5, and some tumours can also locally synthesize and secrete dopamine (DA), forming autocrine and paracrine loops. Through DRD1/5 and DRD2/3/4 receptors, dopamine reshapes multiple intracellular signalling networks and thereby regulates tumour-cell proliferation, apoptosis, autophagy, migration, invasion, EMT, and stemness. Its effects are therefore highly receptor-subtype dependent, cancer-type dependent, and microenvironment dependent, such that dopamine may either promote or suppress tumour progression (Rosas-Cruz et al., 2021). Nevertheless, its net effect across different tumour types and neural-innervation backgrounds still requires systematic evaluation.

Taken together, neurotransmitter-mediated neural regulation should not be interpreted as uniformly tumour-promoting. Instead, its biological output is determined by tumour type, receptor subtype, cellular target, disease stage and the immune-metabolic state of the TME (Amit et al., 2025; Zahalka and Frenette, 2020; Boilly et al., 2017). Cholinergic signalling provides a representative example of this duality: acetylcholine can promote gastric tumorigenesis through M3R-mediated Wnt/EGFR-related signalling and cholinergic-NGF feedback (Zhao et al., 2014; Hayakawa et al., 2017; Yu et al., 2017),whereas enhanced cholinergic-CHRM1 signalling suppresses PDAC progression by attenuating MAPK and PI3K/AKT activation, reducing cancer stem cell-like traits and limiting myeloid inflammation (Renz et al., 2018; Pfitzinger et al., 2020). Similarly, GABAergic signalling shows biphasic effects, with exogenous GABA or GABA-receptor agonists inhibiting CRC-cell proliferation in some *in vitro* settings (S et al., 2016), while elevated GABAergic activity *in vivo* may support tumour invasion, immune remodelling or poor prognosis in CRC and other tumour contexts (Strelez et al., 2025; Barron et al., 2025). Dopamine signalling is also receptor-, dose- and tumour type-dependent: D2R-mediated VEGFR2 internalization can suppress angiogenesis (Basu et al., 2001; Sarkar et al., 2022; Peters et al., 2014), whereas DRD1-or DRD4-related pathways may promote tumour growth, stemness, invasion or treatment resistance in selected cancers (Rosas-Cruz et al., 2021; Yan et al., 2020; Dolma et al., 2016). Adrenergic signalling likewise requires subtype-level resolution, as β2-AR is more closely linked to tumour-cell metabolic reprogramming, NGF induction and myeloid immunosuppression (Kobayashi et al., 2025; Mohammadpour et al., 2021; Dempsey, 2020; Sympathetic nerves suppress T, 2023), whereas β1-AR has been implicated in catecholamine-driven CD8^+^ T-cell exhaustion (Globig et al., 2023; Cox, 2024). These examples emphasize that neurochemical signals should be presented as context-dependent regulatory inputs rather than as uniformly pro-tumoural drivers.

In summary (Table 1), direct regulation mediated by neurotransmitter-receptor signalling is not a single linear pathway, but rather a dynamic network woven together by neural innervation, stromal cells, metabolism, and immunity. Future studies should integrate spatial transcriptomics, spatial proteomics, and single-cell multi-omics to define the spatiotemporal expression patterns and division of labour of key neurotransmitter receptors in tumour cells, and thereby guide the development of more selective neuro-targeted combination strategies (Kobayashi et al., 2025; Komine et al., 2025; Carmi et al., 2025).

**TABLE 1:** Selected cancer-type examples of nerve–TME crosstalk and dominant loop architectures.

Cancer context	Dominant loop architecture	Key pathway(s)	Principal interacting compartments	Mechanistic interpretation and evidence boundary	References
Colorectal cancer	Sympathetic nerve–CAF–tumour feedforward loop; context-dependent GABAergic regulation	NE–ADRB2–NGF/TrkA; ADRA2A/Gi–YAP; GABAergic signalling	Sympathetic nerves, CAFs, CRC cells, Schwann cells	Norepinephrine induces ADRB2-dependent NGF production by stromal cells, thereby increasing sympathetic innervation and catecholamine accumulation. Adrenergic signalling can also activate ADRA2A/Gi–YAP signalling in CRC cells. GABAergic effects should be interpreted as context-dependent rather than uniformly tumour-promoting	Varghese et al. (2025), Tang et al. (2025), Wang et al. (2025b), Kobayashi et al. (2025), Komine et al. (2025), Strelez et al. (2025), Fallon et al. (2023)
Glioma	Neuron-to-glioma synaptic/electrical coupling and tumour-network amplification	AMPAR-dependent neuron–glioma synapses; activity-dependent NLGN3; BDNF–TrkB; tumour microtubes and gap junctions	Excitatory neurons, glioma cells, tumour microtubes, peritumoural neural circuits	Glioma represents one of the strong est examples of direct functional integration between neural circuits and malignant cells. NLGN3 in this row refers to activity-dependent glioma biology	Venkatesh et al. (2019), Venkataramani et al. (2019), Venkatesh et al. (2015), Hausmann et al. (2023), Krishna et al. (2023), Meyer et al. (2024), Taylor et al. (2023), Venkatesh et al. (2017)
Hepatocellular carcinoma	Stress-related sympathetic regulation and context-dependent neuroimmune signalling	β-AR–ERK/CREB; NE-mediated stromal and immune remodelling; ChAT+ CD4^+^ T-cell immunosurveillance	Sympathetic nerves, hepatic stellate cells, TAMs, CD4^+^ T cells, HCC cells	HCC illustrates that neuroimmune signalling may be tumour-promoting or immune-supportive depending on neurotransmitter source, receptor subtype and immune-cell target.	Li et al. (2025a), Dang et al. (2018), Yang et al. (2024), Wang et al. (2024b)
Pan-cancer neural stratification	Neural transcriptional and immune-metabolic coupling patterns	Neural signalling signatures; immune and metabolic feature coupling	Tumour cells, immune cells, stromal cells, neural-associated transcriptional programmes	Pan-cancer analyses support the concept that neural features can stratify tumours according to immune, metabolic and prognostic states, but these data are primarily associative and require tumour-type-specific validation	Gao et al. (2025)
Gastrointestinal cancers/NMSC-associated niche	Schwann-cell-state-specific stromal and immune regulation	NMSC-associated ECM and immune-modulatory programmes	Nonmyelinating Schwann cells, tumour cells, ECM, immune cells	Nonmyelinating Schwann cells should be distinguished from Schwann cells as a broad category. NMSC-related programmes are most appropriately discussed in gastrointestinal and pancreatic cancer contexts rather than as a generic pan-cancer row	Li et al. (2025b), Sun et al. (2023)
Breast cancer	Sensory nerve-driven invasion/metastatic fitness and nerve-to-cancer metabolic support	Sensory nerve–SEMA5A/PlexinB3; neuronal mitochondrial transfer	Sensory nerves, TNBC cells, metastatic breast cancer cells	Sensory nerves can enhance invasive and metastatic behaviour in TNBC. Recent evidence also suggests that neuronal mitochondrial transfer can increase oxidative fitness and metastatic stress tolerance, although generalizability beyond breast cancer remains uncertain	Hoover et al. (2025), Le et al. (2022)
Melanoma	Sensory neuropeptide-mediated immune suppression	Nociceptor-derived CGRP–CALCRL/RAMP1 signalling	Nociceptor neurons, CD8^+^ T cells, myeloid cells	Sensory neuron-derived CGRP can suppress antitumour immunity and promote T-cell exhaustion. This row mainly represents nerve-to-immune regulation rather than a complete bidirectional loop	Balood et al. (2022), Restaino et al. (2024)
Pancreatic cancer	Perineural niche, Schwann-cell immune loop and sensory pseudo-synaptic signalling	NGF/TrkA; CCL2/CCR2; bFGF–IL-33; glutamate–GRIN2D; NMSC-expressed PVT1–kynurenine pathway	PDAC cells, sensory neurons, Schwann cells, NMSCs, TAMs, CAFs, NK cells	PDAC provides a prototypical perineural invasion model in which tumour cells, Schwann cells and immune cells reinforce each other. Sensory neuron–cancer pseudo-synapses and NMSC-mediated immune exclusion should be clearly distinguished	Li et al. (2021), Ni et al. (2023), Renz et al. (2018), Deborde et al. (2016), Deborde et al. (2022), Zhang et al. (2024a), Sun et al. (2023), Ren et al. (2025), Wang et al. (2025c)
Lung cancer	Cholinergic tumour-cell plasticity and tumour–macrophage immune regulation	ACh/M3R/WNT; α5-nAChR/STAT3/Jab1/PD-L1; α5-nAChR/SOX2/CSF-1	Cholinergic signalling pathways, lung cancer cells, TAMs	Cholinergic signalling in lung cancer is mainly represented by drug tolerance, stemness and immune-evasion programmes, and should not be generalized to all tumour types	Nie et al. (2022), Zhu et al. (2022a), Kang et al. (2024)
Neuroblastoma	Neurodevelopmental and neurotrophin-like growth programmes	BDNF–TrkB–PI3K/AKT/MAPK; NLGN3–PI3K/AKT	Neural crest-derived tumour cells, neurotrophin-responsive tumour programmes	NLGN3 here specifically refers to neuroblastoma-cell proliferation through PI3K/AKT activation and should not be conflated with activity-dependent NLGN3 secretion in glioma	Hua et al. (2016), Li et al. (2019)

### 2.3 Indirect regulation of immune, vascular, stromal and metabolic niches

**FIGURE 2:**
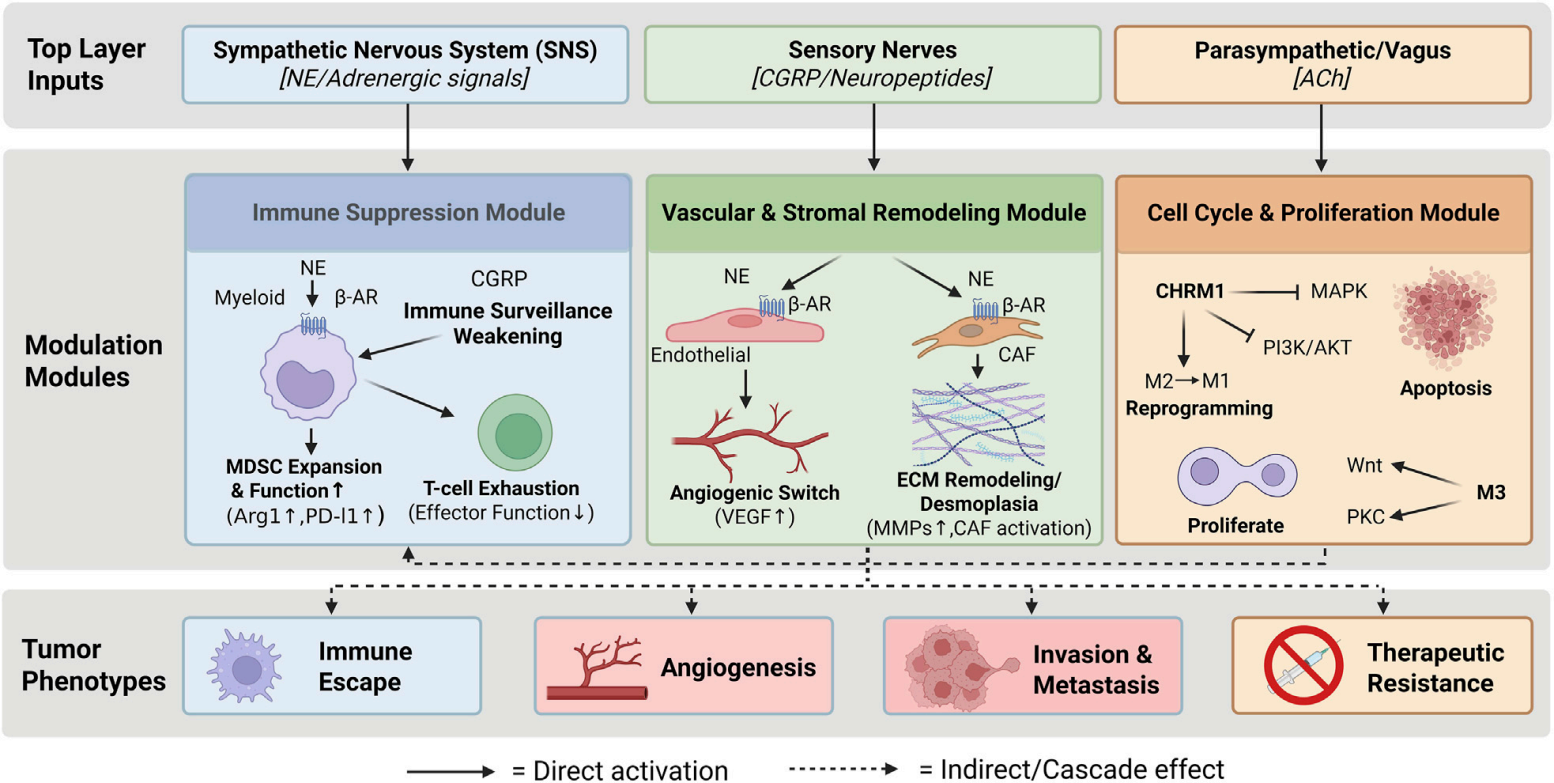
Neural Indirect Regulation Pathways in the TME. This figure illustrates the indirect ways in which neural signals shape the tumour microenvironment (TME). It shows how inputs from the sympathetic, sensory, and parasympathetic nervous systems modulate immune, vascular, and metabolic components, ultimately leading to context-dependent tumour-regulatory outcomes, including tumour-promoting, tumour-suppressive and immune-modulating phenotypes.

Neural regulation of the tumour microenvironment (TME) is not restricted to direct effects on tumour cells themselves; more commonly, it is exerted through non-malignant components of the TME, including immune cells, vascular endothelial cells, fibroblasts, and metabolic niches, thereby forming a plastic dynamic network that drives immune escape, angiogenesis, invasion and metastasis, and metabolic adaptation (Balood et al., 2022; Globig et al., 2023; Cox, 2024; Thaker et al., 2006). In solid tumours, the sympathetic nervous system (SNS) releases norepinephrine (NE) and other stress-related catecholamines, thereby establishing sustained beta-adrenergic signalling within the tumour bed; at the same time, neuropeptides derived from sensory nerves can further ‘reprogramme’ local antitumour immune surveillance through receptor-mediated immunomodulation (Balood et al., 2022; Globig et al., 2023; Thaker et al., 2006). Below, we summarize the key mechanisms and therapeutic implications of these processes from the perspectives of immunosuppression, vascular and stromal remodelling, and metabolic reprogramming (Figure 2).

Neural regulation of immune cells promotes immunosuppression within the TME. For example, norepinephrine (NE) released by the sympathetic nervous system (SNS) can induce immunosuppressive programmes in multiple immune-cell populations within the tumour microenvironment through beta-adrenergic receptors, with particularly prominent effects on the expansion and functional potentiation of myeloid-derived suppressor cells (MDSCs) (Daneshmandi et al., 2024; Cao et al., 2021). Studies have shown that under chronic low-grade stress, sympathetic NE release promotes intratumoural accumulation of MDSCs and activates beta2-AR-dependent transcriptional programmes in these cells, upregulating anti-apoptotic and immunosuppressive molecules such as Arg1, PD-L1, and Bcl-2, thereby enhancing their suppressive function and weakening antitumour immunity (Dempsey, 2020; Sympathetic nerves suppress T, 2023). Consistent with these findings, ADRB1 (beta1-AR) has further been identified as a key node linking sympathetic signalling to CD8^+^ T-cell exhaustion, as sympathetic-catecholaminergic signalling promotes exhaustion-associated phenotypes and functional decline, whereas ADRB1 blockade synergizes with immune-checkpoint blockade (ICB) and improves tumour control (Sympathetic nerves suppress T, 2023; Globig et al., 2023; Cox, 2024). In addition, the inhibitory effects of sensory nerves (nociceptors) on tumour immune surveillance have been observed across multiple models. In solid tumours, CGRP released by sensory nerves acts directly on receptors expressed on immune cells, increases expression of the exhaustion marker PD-1 on T cells, and suppresses IFN-gamma secretion, thereby contributing to an immunosuppressive microenvironment. Neural signalling can also activate downstream JAK/STAT pathways through G-protein-coupled receptors (GPCRs), promoting macrophage polarization towards an M 2 phenotype and further amplifying tumour immune escape (Balood et al., 2022). Thus, neuro-immune cross-talk is not a single pathway, but a networked mode of regulation centred on “myeloid suppression plus T-cell exhaustion,” constituting an important upstream driver of immune escape within the TME (Balood et al., 2022; Dempsey, 2020; Globig et al., 2023).

However, neuroimmune regulation should not be viewed as uniformly immunosuppressive. Immune cells themselves can generate neurotransmitter-like signals; for example, tumour-antigen-driven ChAT+ CD4^+^ T cells support HCC immunosurveillance, and T-cell-specific Chat deletion accelerates hepatocarcinogenesis by compromising antitumour immunity (Zheng et al., 2023). Dopaminergic signalling can also enhance antitumour immunity in selected contexts: dopamine promotes CD103+ tissue-resident memory CD8^+^ T-cell differentiation, increases CD8^+^ TIL accumulation and functionality in CRC models, and DRD5 deficiency impairs intratumoural CD8^+^ T-cell persistence (Chen et al., 2022). In addition, blockade of β-adrenergic signalling, particularly β2-AR, may restore T-cell priming, effector metabolic fitness and exhaustion phenotypes, while also reducing MDSC metabolic fitness and immunosuppressive activity. These findings indicate that neuroimmune signals can exert counter-regulatory or immune-enhancing effects, and that their net consequences depend on neurotransmitter source, receptor subtype, immune-cell target and disease context (Mohammadpour et al., 2021; Qiao et al., 2019; Qiao et al., 2021).

Second, SNS regulation of the vasculature and stroma promotes remodelling of the TME. SNS activation can induce vascular endothelial growth factor (VEGF) expression through NE, enhance endothelial glycolysis and stabilize HIF-1alpha, thereby promoting angiogenesis and tumour perfusion within the TME while concomitantly driving matrix degradation and remodelling (Sun et al., 2025). In ovarian cancer, chronic behavioural stress elevates catecholamine levels within tumours, upregulates VEGF through the ADRB2-mediated cAMP-PKA axis in tumour cells, enhances vascularization, and is accompanied by increased expression of invasion-related factors such as MMP2 and MMP9, thereby increasing tumour burden and promoting a more aggressive growth pattern (Thaker et al., 2006). With respect to tumour invasion and metastasis, beta-AR signalling can also function as a switch by activating key pathways such as Src and thereby accelerating metastasis-associated phenotypes (Armaiz-Pena et al., 2013). At the stromal level, neurogenic beta-AR signalling interacts strongly with fibroblast activation, ECM remodelling, and desmoplasia: on the one hand, ECM remodelling provides tracks for tumour-cell migration and contributes to vascular abnormalization; on the other hand, dense stroma together with abnormal vasculature impedes drug delivery and creates an immune-excluded microenvironment, thereby diminishing responses to radiotherapy, chemotherapy, and immunotherapy (Thaker et al., 2006; Yaman et al., 2022). Accordingly, the ‘nerve-vascular-stroma’ axis may be regarded as an important source of structural therapy resistance within the TME (Thaker et al., 2006; Armaiz-Pena et al., 2013; Yaman et al., 2022).

Neurotransmitters also influence metabolic reprogramming within the acidic TME. Stress-related catecholamines and other neurotransmitters affect tumour-cell metabolism directly and also indirectly shape an immunosuppressive microenvironment by altering local nutrient availability and metabolic by-product profiles. In terms of glucose metabolism, mediators such as NE promote tumour glucose and lipid metabolism and enhance the Warburg effect; in addition, chronic stress activates the beta2-AR/PKA/CREB1 pathway, upregulates GLUT 1 and key glycolytic enzymes, and increases lactate production, whereas lactate accumulation has been recognized as a suppressor of effector T-cell function and a promoter of immunosuppression (Guan et al., 2023). With respect to lipid metabolism, tumour-infiltrating MDSCs often exhibit increased lipid uptake and enhanced fatty-acid oxidation (FAO), and FAO inhibition can weaken their immunosuppressive activity and improve antitumour immunity (Hossain et al., 2015). Given that the SNS can systemically regulate lipid mobilization and local metabolic substrate availability, neural signalling is likely to further “feed” immunosuppressive cell populations and amplify immune escape by altering lipid supply and oxidative preferences (Sun et al., 2025; Hossain et al., 2015; Al-Khami et al., 2017). Overall, neural regulation and metabolic adaptation are tightly intertwined, allowing the TME to maintain a pro-tumour steady state characterized by high glycolysis, high lactate, and a shift in lipid metabolism, thereby increasing the risks of therapy resistance and recurrence (Sun et al., 2025; Guan et al., 2023; Hossain et al., 2015; Al-Khami et al., 2017).

Recent work has uncovered an additional metabolic dimension of nerve–tumour communication. Multiple experimental platforms, including breast cancer animal models, nerve–tumour co-culture systems, MitoTRACER lineage tracing technology and primary human breast tumour specimen analysis, provide evidence that cancer-associated neurons transfer mitochondria to neighbouring malignant cells, with tunnelling nanotube-like intercellular conduits acting as one primary transport route (Hoover et al., 2025). Within this experimental framework, breast cancer cells that acquire neuronal mitochondria display stronger mitochondrial respiratory activity, elevated ATP synthesis, superior redox-buffering capacity, pronounced stem cell-like traits, and improved survival against oxidative and fluid shear stress. Of note, baseline invasive potential of these cells was unchanged under *in vitro* culture conditions (Hoover et al., 2025). *In vivo* lineage tracking data further reveal that tumour cells which have taken up neuron-derived mitochondria, alongside their descendant populations, preferentially accumulate at distant metastatic lesions in preclinical tumour models (Hoover et al., 2025). Collectively, these observations imply that intercellular mitochondrial transfer from neurons to cancer cells facilitates metabolic plasticity and enables tumour cells to withstand metastatic stressors within specific tumour contexts, most notably breast cancer experimental systems. Nevertheless, it remains unclear whether this phenomenon occurs broadly across diverse cancer subtypes, and further research is required to validate any potential translational utility for therapeutic intervention.

In light of the above neural influences on the tumour microenvironment (Table 2), neuro-targeted strategies are increasingly viewed as complementary to existing therapies (Zahalka and Frenette, 2020; Boilly et al., 2017; Kobayashi et al., 2025; Renz et al., 2018). For example, beta-blockers provide a direct entry point for intervention in neural signalling. On the one hand, blockade of the ADRB1/ADRB2 axis may simultaneously alleviate T-cell exhaustion and myeloid suppression, thereby reducing immunosuppression within the TME from an upstream level (Dempsey, 2020; Globig et al., 2023). On the other hand, beta-blockers may under certain conditions improve stress-related angiogenic pathways and exhibit mechanistic complementarity with ICB (Sympathetic nerves suppress T, 2023; Thaker et al., 2006; Armaiz-Pena et al., 2013; Hossain et al., 2015). Particularly in the era of immunotherapy, β-adrenergic blockade provides a mechanistically plausible but clinically unconfirmed adjunctive strategy. Preclinical and translational studies suggest that β-adrenergic signalling can promote myeloid suppression, CD8^+^ T-cell dysfunction and stress-related angiogenic programmes, whereas a phase I propranolol–pembrolizumab study in metastatic melanoma demonstrated safety, tolerability and preliminary activity (Kokolus et al., 2018; Gandhi et al., 2021). However, aggregated clinical evidence remains mixed: recent meta-analytic data based largely on non-randomized studies did not confirm a consistent OS or PFS benefit from adding β-blockers to ICIs, and substantial heterogeneity was observed across cancer types, β-blocker classes and treatment contexts (Tokunaga et al., 2025; Zhang et al., 2025). Therefore, β-blocker–ICI combinations should currently be regarded as investigational and hypothesis-generating rather than ready for routine precision-oncology implementation.

**TABLE 2:** Potential neuro-targeted agents, mechanisms and evidence source/type.

Agent/class	Pharmacological definition	Proposed cancer-neuroscience rationale	Cancer context	Evidence level/translational status	References
β-adrenergic blockers, propranolol or subtype-selective β-blockers	β-AR antagonists; propranolol is a non-selective β1/β2 blocker	May reduce adrenergic stress signalling, angiogenic programmes, myeloid suppression and T-cell dysfunction	CRC, melanoma, prostate cancer, HCC and other stress-responsive tumours	Preclinical mechanistic evidence; retrospective or observational clinical associations; phase I propranolol–pembrolizumab feasibility data in melanoma; pooled clinical evidence remains mixed	Varghese et al. (2025), Mohammadpour et al. (2021), Komine et al. (2025), Cole and Sood (2012), Globig et al., 2023; Cox (2024), Kokolus et al. (2018), Gandhi et al. (2021), Zhang et al. (2025)
GABAergic pathway modulators	Includes GABA_B receptor-related modulation, GABA-metabolic interventions and GABAergic synapse-targeting approaches. Diazepam-like benzodiazepines are GABA_A receptor positive allosteric modulators, not direct GABA agonists	GABA_B receptor activation may suppress CRC proliferation or EMT in some models, whereas GABA metabolism and GABAergic neuron–tumour synapses may support glioma biology in other contexts	CRC, glioma, diffuse midline glioma	Mainly *in vitro*, organoid or preclinical mechanistic evidence; strongly context-dependent	Tang et al. (2025), Hujber et al. (2018), Huang et al. (2022), Strelez et al. (2025), Barron et al. (2025)
SERT inhibitors/SSRIs, sertraline or fluoxetine	Serotonin transporter inhibitors that increase extracellular or intratumoural serotonin availability	May modulate tumour immunity through SERT-dependent regulation of intratumoural serotonin and CD8^+^ T-cell function	Solid-tumour immune contexts; epidemiological signals in breast, prostate or bladder cancer should be interpreted separately	Emerging mechanistic and translational evidence; not established as standard anticancer therapy	Ashbury et al. (2010), Liu et al. (2020), Li et al. (2025c)
Serotonin receptor-targeted strategies	Pharmacological modulation of specific 5-HT receptors, such as HTR2B, HTR2A or HTR1D	May affect tumour-cell proliferation, EMT, metastasis, stemness or metabolic signalling depending on receptor subtype and cancer context	CRC, HCC and selected immune or metabolic contexts	Preclinical and mechanistic tumour-cell evidence; receptor-specific and cancer-context-dependent	Li et al. (2024), Carmi et al. (2025), Peters et al. (2014), Zhu et al. (2022b), Soll et al. (2010)
TRK inhibitors, larotrectinib or entrectinib	TRK kinase inhibitors for NTRK fusion-positive tumours	Target oncogenic TRK fusion signalling rather than NGF ligand signalling or tumour innervation	NTRK fusion-positive solid tumours	Approved molecular targeted therapy for biomarker-selected NTRK fusion cancers	Hou et al. (2024)
NGF/TrkA-axis blockade and anti-NGF strategies	Interventions targeting NGF ligand, NGF–TrkA signalling or NGF-mediated neural remodelling	May reduce tumour-associated neural sprouting, perineural invasion or cancer pain	PDAC PNI models; bone metastasis pain settings	Preclinical anti-axonogenesis/PNI rationale; randomized evidence for analgesic efficacy and safety in bone metastasis pain; direct antitumour benefit not established	Aloe et al. (2016), Micera et al. (2007), Pundavela et al. (2015), Hayakawa et al. (2017), Fallon et al. (2023)
AMPA receptor antagonists, perampanel	Non-competitive AMPAR antagonist	May weaken AMPAR-dependent neuron–glioma synaptic input and reduce neuronal activity-driven tumour support	Glioma/glioblastoma; glioma-associated seizures	Strong preclinical rationale; established antiseizure use; PerSurge/NOA-30 phase II evaluates perioperative perampanel in recurrent/progressive glioblastoma	Venkatesh et al. (2019), Venkataramani et al. (2019), Heuer et al. (2024)
CGRP/CALCRL–RAMP1 pathway blockade	Neuropeptide-axis inhibition	May relieve nociceptor-driven immune suppression and T-cell exhaustion in selected tumours	Melanoma, HNSCC and possibly other sensory nerve-rich tumours	Preclinical neuroimmune evidence	Balood et al. (2022), Darragh et al. (2024), Wang et al. (2025c), Restaino et al. (2025)

In the future, spatial transcriptomics, spatial proteomics, single-cell multi-omics, and neuroanatomical markers may be used to resolve the spatiotemporal coupling among nerve-fibre distribution, neurotransmitter gradients, and immunometabolic states, thereby enabling more precise neuro-targeted stratification and rational combination-treatment design across different cancer types and molecular subgroups (Li W. et al., 2025; Balood et al., 2022; Globig et al., 2023; Yaman et al., 2022).

## 3 Feedback from the tumour microenvironment to the nervous system

### 3.1 TME-induced neuroplasticity and tumour innervation

**FIGURE 3:**
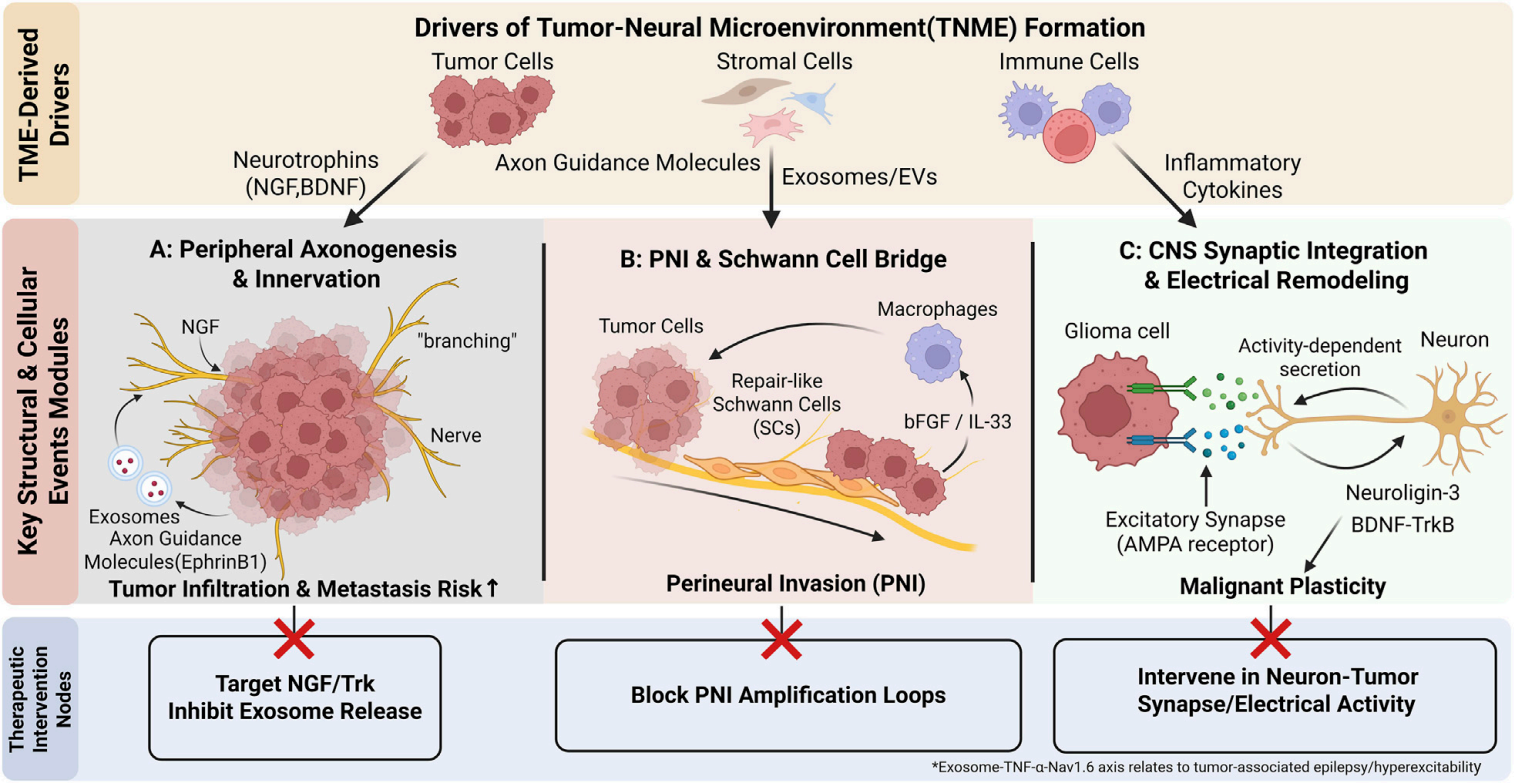
TME-Induced Neuroplasticity and TNME Formation Mechanisms and Intervention Nodes This diagram illustrates how the tumour microenvironment actively induces neuroplasticity, leading to the formation of a tumour-neural microenvironment (TNME). It details processes like axonogenesis and perineural invasion, and identifies potential therapeutic targets to disrupt these interactions.

Feedback regulation exerted by the tumour microenvironment (TME) on the nervous system is a critical component of bidirectional nerve-tumour cross-talk. Increasing evidence indicates that tumour cells, stromal cells, and immune cells within the TME can drive structural and functional plastic changes (neuroplasticity) in the peripheral and central nervous systems by secreting neurotrophic factors, axon guidance molecules, extracellular vesicles, and inflammatory and metabolic mediators. These changes include axonogenesis, neural infiltration, glial-cell reprogramming, and remodelling of neural electrical activity, thereby generating a tumour neuro-microenvironment (TNME) with distinct biological features in the tumour bed. The TNME is not a passive accompaniment of neural components; rather, it often forms positive-feedback circuits with tumour cells that promote invasion, metastasis, symptom burden, and therapeutic resistance, and has driven the emergence of a research framework centred on a “nerve-dependent tumour ecosystem” (Figure 3).

In peripheral solid tumours, the neurotrophic factor-receptor axis is one of the core pathways driving neural remodelling. Tumour cell-derived nerve growth factor (NGF) is associated with the appearance of intratumoural axons and with increased risk of lymph-node metastasis, and tumour-induced neurite outgrowth can be partially inhibited by NGF-neutralizing antibodies, supporting a potential causal chain of “tumour-secreted NGF - axonogenesis - tumour invasion/metastasis” (Pundavela et al., 2015). In the gastrointestinal stem-cell niche, nerves help regulate the dynamics of normal and malignant stem cells. Cholinergic signalling associated with tuft cells and nerves can upregulate epithelial NGF expression, thereby promoting enteric neural expansion and tumour development, whereas blockade of NGF/Trk signalling or elimination of key cholinergic cells suppresses tumour progression, suggesting that the “cholinergic-NGF” axis may represent a potential target for tumour prevention and treatment (Hayakawa et al., 2017). Tumour-derived exosomes are not passive by-products within the tumour neural microenvironment (TNME), but rather important upstream signal carriers that drive tumour innervation. In head and neck squamous cell carcinoma (HNSCC), patient-derived tumour exosomes markedly induce neurite outgrowth in PC12 cells; meanwhile, newly formed nerves within tumour tissues are mainly represented by beta-III tubulin-, Tau-, and TRPV1-positive fine fibres with a sensory nerve-like phenotype, suggesting that tumours can actively recruit local neural components through exosome-mediated axonogenesis (Madeo et al., 2018). More importantly, either reducing exosome release or pharmacologically blocking it with the neutral sphingomyelinase inhibitor GW 4869 markedly decreases the degree of tumour innervation and expression of neural markers, functionally demonstrating that exosomes are important mediators of tumour neuralization (Vermeer, 2019). Mechanistically, axon guidance molecule EphrinB1 carried by exosomes significantly enhances their axonogenic activity and promotes formation of fine tumour-associated sensory nerve branches. In addition, TP53 loss can further reshape the miRNA repertoire of tumour-derived extracellular vesicles (EVs), thereby promoting sensory axon extension and reprogramming them towards a pro-tumour adrenergic phenotype (Amit et al., 2020; Restaino et al., 2024). Similar exosome-mediated neural recruitment has also been observed in HPV-related tumours such as cervical cancer, suggesting that the axis of ‘tumour exosomes/sEVs - axon guidance molecules and miRNA cargo - axonogenesis/neural reprogramming - tumour innervation’ likely constitutes a key molecular programme driving TNME formation and its continued remodelling (Lu et al., 2019; Guo and Gil, 2022).

At the cellular level, Schwann cells (SCs) and their “repair-like” reprogramming are considered a critical hub connecting neural remodelling with tumour invasion (Deborde et al., 2016). Increasing evidence indicates that tumours can hijack the repair programme initiated by SCs after nerve injury, converting them from a relatively homeostatic mature phenotype into an activated state characterized by high plasticity, pro-migratory behaviour, and regenerative capacity, thereby actively shaping a microenvironment conducive to cancer-cell spread along nerves (Deborde et al., 2016; Deborde et al., 2022; Kruglov et al., 2023). In models of perineural invasion (PNI), direct contact between SCs and cancer cells induces the formation of SC-oriented protrusions in cancer cells, promotes cancer-cell dissociation and migration towards nerves, and markedly enhances invasion through three-dimensional matrices. This effect depends on heterotypic cell-cell contact rather than soluble factors alone and is mediated in part by SC-expressed NCAM 1. Subsequent studies further showed that cancer cells can induce c-Jun-dependent reprogramming in SCs, generating tumour-activated Schwann cell tracks (TAST). Functionally resembling the Bungner bands observed during injured-nerve repair, these dynamically aligned SC tracks provide a physical guidance scaffold for cancer cells, enhance their migration and invasiveness, and are associated with poorer survival in patients with PDAC 68. Meanwhile, in melanoma, peritumoural SCs can enter a transcriptional state with repair-like features, suggesting that repair-like SC reprogramming is not restricted to a single tumour type but may represent a broader mechanism of tumour neural niche remodelling (Kruglov et al., 2023). On this basis, neuropeptides and inflammatory factors can further amplify the pro-invasive phenotype of SCs. In cervical cancer, tumour cells secrete neuromedin-B (NMB), which acts on NMBR expressed on SCs, opens T-type calcium channels, and induces Ca2+ influx through PKA signalling, thereby driving morphological and transcriptomic reprogramming of SCs, enhancing their proliferation, migration, CCL2 secretion, and axonal regeneration, and ultimately promoting PNI; accordingly, combined serum NMB and CCL2 levels have been proposed as potential markers for PNI-risk assessment and decisions regarding nerve-sparing surgery (Gao et al., 2024). In PDAC, tumour-associated macrophages (TAMs) can activate SCs through the bFGF/PI3K-Akt/c-Myc/GFAP axis (Zhang B. et al., 2024), whereas activated SCs subsequently secrete IL-33, recruit macrophages, and promote their polarization towards a pro-tumour phenotype, thereby forming a bFGF/IL-33 positive-feedback loop that drives PNI progression; correspondingly, high CD (Deborde et al., 2022) or GFAP expression is associated with a higher incidence of PNI and worse 5-year survival, suggesting that immune cell-SC interactions are an important mechanism maintaining the perineural invasive niche (Zhang B. et al., 2024). Overall, SCs are not passive neural support cells in PNI, but effector cells that can be persistently educated and actively reprogrammed by tumours and inflammatory signals. Through direct guidance by cell contact, construction of repair-like tracks, and paracrine interactions with immune cells, they jointly promote neural remodelling, cancer-cell dissemination along nerves, and worsening of the tumour neural microenvironment.

Compared with peripheral solid tumours, in which the tumour neural microenvironment is dominated by perineural invasion, Schwann-cell reprogramming, and axonal regeneration, TNME in CNS tumours, particularly diffuse gliomas, is characterized more prominently by deep integration with local neural circuits at the levels of electrical activity and synapses (Venkatesh et al., 2019; Venkataramani et al., 2019). Previous studies have shown that glioma cells are not merely passively exposed to the neurotransmitter milieu, but can form glutamatergic synapse-like connections with excitatory neurons and receive AMPA receptor-dependent excitatory input as postsynaptic elements (Venkatesh et al., 2019; Venkataramani et al., 2019). On this basis, neuronal activity can also induce non-synaptic extracellular potassium currents, and these depolarizing signals are further amplified through a network composed of gap junctions and tumour microtubes (TMs) between tumour cells, thereby generating an electrophysiologically integrated state; blocking this electrochemical communication suppresses growth of glioma xenografts and prolongs survival (Hausmann et al., 2023). Furthermore, gliomas do not simply receive neural regulation unidirectionally, but can in turn remodel host neural circuits. Studies in human glioblastoma have shown that tumour regions with high functional connectivity are enriched for tumour-cell subpopulations with greater integrative capacity, invasiveness, and proliferative potential, and are associated with worse survival and impaired language function, indicating that gliomas can substantially alter higher cortical function by strengthening their coupling with brain networks (Krishna et al., 2023). At the same time, evidence suggests that glioma-associated circuit remodelling is frequently accompanied by increased excitability in the peritumoural cortex (Venkatesh et al., 2019); glioblastoma can induce sustained cortical hyperexcitability from early stages of disease, and patients may present with phenotypes ranging from cognitive impairment to epileptic seizures, providing a neural-network-level explanation for glioma-associated epilepsy (Meyer et al., 2024). In addition to direct electro-synaptic integration, activity-dependent secreted factors are also key pro-tumour signals in the central TNME. Among them, Neuroligin-3 (NLGN3) is currently one of the most representative neuron-derived tumour-promoting factors: neuronal activity induces NLGN3 release, and NLGN3 markedly promotes high-grade glioma-cell proliferation while activating downstream growth pathways such as PI3K-mTOR; under conditions of NLGN3 deficiency or blockade of its release, glioma growth is markedly suppressed, indicating that this molecule is not only an important bridge linking neuronal activity to tumour growth but also a clearly actionable target (Venkatesh et al., 2015). Beyond this, gliomas can hijack the activity-dependent synaptic plasticity programme of the normal nervous system to generate so-called malignant plasticity: brain-derived neurotrophic factor (BDNF), acting through TrkB receptors on glioma cells, enhances AMPA-receptor trafficking to the cell surface, increases glutamate-evoked current amplitude, and raises the number of neuron-glioma synapses, thereby amplifying tumour responsiveness to neuronal activity and accelerating tumour progression. In multiple paediatric high-grade glioma and DIPG models, genetic or pharmacological blockade of TrkB markedly suppresses tumour growth and prolongs survival (Taylor et al., 2023). Therefore, for CNS tumours such as glioma, the TNME should no longer be understood simply as a unidirectional paracrine environment in which neurotransmitters influence tumours, but rather as a dynamic nerve-tumour functional consortium sustained jointly by neuron-glioma synaptic communication, tumour networks, and activity-dependent signals such as NLGN3 and BDNF-TrkB. This framework also provides a theoretical basis for therapeutic development targeting the AMPA receptor, gap-junction-coupled tumour microtubes, NLGN3 shedding, and TrkB signalling (Taylor et al., 2023).

From the perspective of translational medicine, the actionable nodes within the TNME can also be broadly categorized into three groups. First, blockade of tumour-induced neuralization and axonogenesis. Neurotrophic axes such as NGF-TrkA not only participate in tumour-associated neural recruitment but also enhance the capacity of tumour cells to invade along nerves; in pancreatic cancer, NGF blockade significantly reduces the ability of tumour cells to invade nerves. Meanwhile, tumour-derived exosomes can serve as important mediators of axon guidance and neural remodelling; small extracellular vesicles carrying EphrinB1 promote neurite outgrowth, and inhibition of exosome release with GW 4869 markedly reduces beta-III tubulin-positive nerve fibres within tumours, underscoring the translational potential of suppressing exosome-mediated neural recruitment (Hayakawa et al., 2017; Madeo et al., 2018). Second, targeting the pro-PNI circuit jointly formed by Schwann cells and immune cells to interrupt the hijacking of a nerve-repair-like stroma. SC-derived CCL2 promotes proliferation, migration, invasion, and epithelial-mesenchymal transition in cervical cancer cells through CCR 2, whereas in PDAC, tumour-associated macrophages (TAMs) secrete bFGF to activate SCs, which in turn secrete IL-33 to recruit macrophages and induce their polarization towards a pro-tumour phenotype, thereby generating a bFGF-IL-33 positive-feedback loop that sustains PNI (Cox, 2024; Zhang B. et al., 2024). Third, interference with neuron-tumour synapses and electrical activity. High-grade gliomas can receive AMPA receptor-dependent synaptic input from neurons and promote tumour proliferation and invasion through membrane depolarization and network amplification. BDNF-TrkB signalling enhances the plasticity of these neuron-glioma synapses. Based on this mechanism, major translational objectives of AMPA receptor antagonists include assessing changes in neuron-tumour synaptic connectivity within tumour tissue and short-term preoperative tumour growth rates, thereby testing the clinical feasibility of the therapeutic concept of weakening nerve-tumour connectivity (Venkatesh et al., 2019; Heuer et al., 2024; Taylor et al., 2023). It should be emphasized that some neurotrophic factor-targeted drugs are still used mainly for management of neurological symptoms rather than for validation of antitumour efficacy. For example, in a randomized, placebo-controlled phase III trial of the anti-NGF antibody tanezumab for pain from bone metastases, the primary end points remained analgesic efficacy and safety; although the primary pain end point was reached at 8 weeks, direct evidence for tumour control, delayed recurrence, or survival benefit remains insufficient (Fallon et al., 2023). Future studies should therefore define the heterogeneity of the TNME across cancer types, stages, and treatment pressures, and on this basis develop multidimensional combination strategies. For example, inhibiting glutamatergic neuron-glioma interactions can remodel TAM states and alleviate local immunosuppression, providing a new biological rationale for synergy between neuro-targeted therapies and immunotherapy (Nejo et al., 2025).

### 3.2 Dynamic feedback loops linking nerves and the TME

**FIGURE 4:**
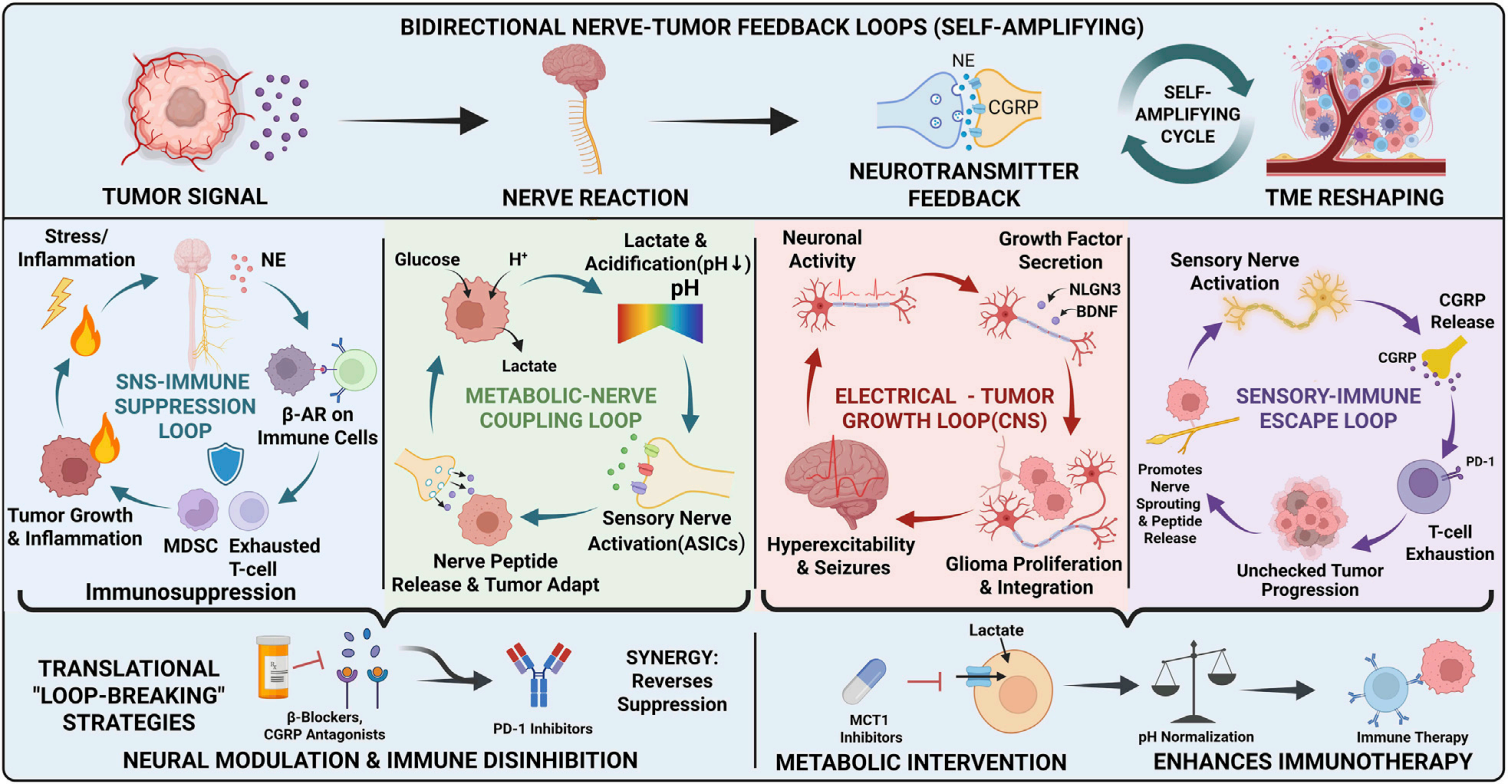
Bidirectional Interactive Dynamic Loops and Translational “Loop-Breaking” Strategies This final figure visualizes the dynamic, self-reinforcing feedback loops between the nervous system and the tumour. It categorizes these loops and proposes translational “loop-breaking” strategies, such as combining neural modulation with immunotherapy, to disrupt these cancer-promoting cycles.

Interactions between the tumour microenvironment (TME) and the nervous system are not unidirectional stimuli, but rather a multilayered dynamic network composed of ‘tumour-derived signals - neural sensing and integration - neurotransmitter/neuropeptide feedback to the TME’ (Monje et al., 2020). Increasing evidence indicates that neural components are not only important constituents of the TME, but can also influence tumour initiation and progression through local innervation, systemic stress responses, and sustained neuro-immune regulation; conversely, tumours and their immune-stromal components can remodel neural activity and neural phenotypes, thereby forming local and even systemic feedback loops that continuously shape the tumour niche and promote immune escape, angiogenesis, stromal remodelling, metabolic adaptation, and impaired therapeutic responses (Winkler et al., 2023; Pu et al., 2025; Ma and Kroemer, 2024). This framework suggests that tumours should be viewed more appropriately as systemic pathological units embedded within a neuro-immune-metabolic axis, and that identifying closed-loop structures and actionable nodes within this axis is an important prerequisite for developing multitarget combination strategies (Figure 4).

Among these feedback circuits, the sympathetic-immunosuppressive loop represents a relatively clear pathway with realistic translational potential. Local tumour inflammation, nociceptive input, and stress-related signals can be sensed and integrated by peripheral nerves and the CNS, and can then be relayed back to the TME through sympathetic output, thereby enhancing local norepinephrine (NE) and adrenergic signalling; NE in turn activates cAMP/PKA and related downstream pathways through beta-adrenergic receptors, promoting tumour-cell invasion, angiogenesis, and adaptive survival while simultaneously shaping stromal and immune environments conducive to tumour progression (Cole and Sood, 2012; Thaker et al., 2006; Sood et al., 2006). At the same time, NE can upregulate molecules such as MMP-2, MMP-9, and VEGF, thereby enhancing tumour-cell invasiveness and angiogenic capacity.

The metabolic-neural loop provides another important amplification axis for the tumour niche. Lactate accumulation and local acidification resulting from enhanced tumour glycolysis are not merely by-products of metabolic reprogramming, but active signalling events that can reshape the tumour microenvironment (Colegio et al., 2014; Wang et al., 2020). Studies have shown that lactate can directly suppress activation (Wemmie et al., 2013), cytokine production, and survival of effector T cells and NK cells, while driving macrophages towards an HIF-1alpha-dependent immunosuppressive and pro-angiogenic phenotype, thereby reinforcing tumour immune escape (Colegio et al., 2014; Wang et al., 2020; Wemmie et al., 2013; Brand et al., 2016). At the same time, the acidic microenvironment can activate or sensitize peripheral nociceptors through proton-sensitive channels such as ASICs and TRPV 1, thereby amplifying pain input; among these, TRPV1-mediated Ca2+ influx is more directly associated with release of neuropeptides such as CGRP, whereas ASIC 3 is more involved in sensing acid stimuli and increasing neuronal excitability (Yoneda et al., 2015; Boillat et al., 2014). The axes of “lactate acidification-immunosuppression” and “acid-sensitive nociception-neuropeptide feedback” are likely superimposed within the same spatial niche, together forming a feedback circuit jointly driven by metabolism and neural signalling.

In CNS tumours, especially high-grade gliomas, this loop manifests even more directly as an axis of “neuronal activity-tumour growth.” Classical studies have shown that Neuroligin-3 (NLGN3), released in response to neuronal activity, markedly promotes glioma-cell proliferation and activates growth pathways such as PI3K-mTOR; high-grade gliomas display a clear dependence on NLGN3, indicating that it is not only a paracrine growth signal but also a potential therapeutic target (Venkatesh et al., 2015; Venkatesh et al., 2017). In addition to soluble factors, glioma cells can receive glutamatergic input via AMPA receptor-dependent neuron-glioma synapses, thereby becoming functionally integrated into host neural circuits (Venkatesh et al., 2019; Venkataramani et al., 2019). On the other hand, glutamate export by glioma cells through system xc-disrupts peritumoural glutamate homeostasis, induces neuronal hyperexcitability and seizure-like activity, and forms a pathological positive-feedback loop of “increased excitability-enhanced tumour adaptation and invasion” (Buckingham et al., 2011). In peripheral solid tumours, tumour-derived small extracellular vesicles can also reprogramme TRPV1+ sensory neurons, promoting nascent protein translation, calcium influx, and pain sensitization, suggesting that vesicular signalling may likewise serve as an important wireless channel for tumour-neural communication (Inyang et al., 2024). Regarding the neuro-immune loop, sensory neuron-derived neuropeptides provide a direct mechanism linking neural input to immune escape. In melanoma models, tumour cells induce increased neurite outgrowth, enhanced responsiveness to noxious stimuli, and increased neuropeptide release in nociceptor neurons; CGRP then promotes exhaustion of tumour-infiltrating CD8^+^ T cells and impairs immune surveillance through its receptor pathway, thereby accelerating tumour progression (Balood et al., 2022). Similar phenomena have also been observed in head and neck squamous cell carcinoma, in which CGRP released by sensory nerves suppresses tumour-infiltrating lymphocytes and promotes tumour growth, further supporting the view that CGRP is a key effector molecule in tumour neuro-immune interactions (Darragh et al., 2024).

From a translational standpoint, strategies to disrupt these feedback loops can be summarized as neuromodulation, immune disinhibition, metabolic intervention, and their combinations. Beta-blockers have attracted attention because of their established non-oncological safety profiles and strong mechanistic rationale; however, their use as adjuncts to ICIs remains investigational. In a prospective phase I study, the non-selective beta-blocker propranolol combined with the PD-1 inhibitor pembrolizumab showed feasibility and preliminary signals of activity in metastatic melanoma (Gandhi et al., 2021), whereas recent pooled clinical analyses have not established a consistent survival benefit (Tokunaga et al., 2025; Zhang et al., 2025). Metabolic interventions aimed at feedback circuits, including inhibition of lactate production or transport, could in principle alter tumour metabolic dependence and the acidification-associated immunosuppressive milieu, and may therefore complement immunotherapy (Halford et al., 2023). Given the redundancy and compensatory pathways inherent to feedback-loop networks, future work will need to combine spatial omics and single-cell approaches with functional imaging to resolve the spatiotemporal coupling among nerve-fibre distribution, immune-cell states, and metabolic gradients, and to design multimodal combination therapies supported by mechanistic models (Monje et al., 2020).

## 4 Molecular and cellular principles of bidirectional regulation

Reverse feedback from the tumour microenvironment (TME) to the nervous system represents an important functional module of tumour-host interactions. Existing evidence indicates that peripheral tumours and their associated inflammation can influence the central nervous system through multiple routes, including cytokines, the vascular endothelium-blood-brain barrier (BBB) interface, circumventricular organs, and resident immune cells in the brain (Table 3). Among these, dynamic responses at the level of microglia and the cerebrovascular interface may constitute early amplification steps through which peripheral inflammatory signals trigger central neuroinflammation. Even in the absence of CNS involvement, tumours can induce fatigue, affective disturbances, and cognitive impairment before treatment, and treatment-related inflammation can further amplify this process, ultimately leading to neurobehavioural phenotypes such as pain, cancer-related fatigue, and cancer-related cognitive impairment (CRCI). In other words, inflammatory mediators released by the TME not only shape a local immunosuppressive niche but can also remodel the host in reverse via a “peripheral inflammation-brain response-neuroendocrine and immune feedback” axis, thereby forming a systemic closed loop that favours sustained tumour progression (Scheff and Saloman, 2021; Mampay et al., 2021; Demos-Dav et al., 2024).

**TABLE 3:** Context-dependent neurotransmitter and neuropeptide signalling in nerve–TME crosstalk.

Neurotransmitter/neuropeptide	Major receptor or transporter context	Principal cellular targets	Representative tumour-regulatory effects	Required caveat	References
Norepinephrine/epinephrine	β2-AR, β1-AR, ADRA2A	Tumour cells, CAFs, endothelial cells, MDSCs, CD8^+^ T cells	β2-AR signalling can promote NGF-associated neuralization, tumour-cell metabolic adaptation, angiogenesis and myeloid immunosuppression. β1-AR has been implicated in CD8^+^ T-cell exhaustion. ADRA2A/Gi can activate YAP in CRC cells	Adrenergic signalling should be resolved by receptor subtype, cellular target, stress context and disease stage	Kobayashi et al. (2025), Mohammadpour et al. (2021), Komine et al. (2025), Cole and Sood (2012), Dempsey (2020), Globig et al., 2023; Cox (2024), Thaker et al. (2006), Guan et al. (2023), Dang et al. (2018), Yang et al. (2024), Cui et al. (2019)
GABA	GABA_A receptor, GABA_B receptor, GABA-metabolic enzymes such as SSADH	Tumour cells, glioma cells, neuron–tumour synapses	GABA_B receptor activation can suppress CRC proliferation or EMT in some models, whereas GABAergic neuron–glioma synapses and GABA metabolism may support glioma biology	Do not generalize GABA as uniformly tumour-suppressive or tumour-promoting. Effects depend on receptor subtype, tumour type and multicellular context	Tang et al. (2025), Hujber et al. (2018), Huang et al. (2022), Strelez et al. (2025), Barron et al. (2025)
Glutamate	AMPAR, NMDAR/GRIN2D, system x_c−	Glioma cells, PDAC cells, neurons	AMPAR-dependent synapses support glioma growth and invasion. GRIN2D-type pseudo-synapses have been reported in PDAC. Glioma-derived glutamate can increase neuronal hyperexcitability and seizure-like activity	CNS glioma synapses and extracranial pseudo-synapses should be distinguished mechanistically and therapeutically	Venkatesh et al. (2019), Venkataramani et al. (2019), Taylor et al. (2023), Buckingham et al. (2011), Ren et al. (2025)
Acetylcholine	mAChRs including M3R and CHRM1; nAChRs including α5-nAChR	Gastric epithelial/tumour cells, PDAC cells, lung cancer cells, macrophages, CD4^+^ T cells	M3R signalling can promote gastric tumorigenesis and colon-cancer proliferation; α5-nAChR-related signalling can promote lung cancer drug tolerance, stemness and immune escape; CHRM1-related cholinergic signalling suppresses PDAC growth and inflammation; ChAT+ CD4^+^ T cells can support HCC immunosurveillance	Acetylcholine signalling is strongly context-dependent. This duality should be mirrored in Figure 1 and its legend	Zhao et al. (2014), Renz et al. (2018), Larabee et al. (2022), Hayakawa et al. (2017), Pfitzinger et al. (2020), Nie et al. (2022), Zhu et al. (2022a), Kang et al. (2024)
CGRP	CALCRL/RAMP1	CD8^+^ T cells, tumour-infiltrating lymphocytes, myeloid cells, CAF-associated immune niches	Nociceptor-derived CGRP can promote CD8^+^ T-cell exhaustion and impair antitumour immunity in melanoma and HNSCC models. In PDAC, sensory neuron-related CGRP signalling has been linked to CAF–NK-cell immune regulation	Strongest evidence supports sensory neuroimmune suppression; clinical translation remains early	Balood et al. (2022), Restaino et al. (2024), Darragh et al. (2024), Wang et al. (2025c)
Dopamine	D1-like receptors: DRD1/DRD5; D2-like receptors: DRD2/DRD3/DRD4	Endothelial cells, tumour cells, glioma stem-like cells, CD8^+^ T cells	D2R-related signalling can suppress VEGFR2-driven angiogenesis. DRD1 can be tumour-promoting in HCC. DRD4 supports glioblastoma stem-like cell survival. DRD5 signalling can promote CD8^+^ tissue-resident memory differentiation and antitumour immunity	D1, D2, D4 and D5 effects should not be merged. Tumour-cell, endothelial and immune effects may diverge	Basu et al. (2001), Sarkar et al. (2022), Peters et al. (2014), Rosas-Cruz et al. (2021), Yan et al. (2020), Dolma et al. (2016)
Serotonin/5-HT	5-HT receptors including HTR2B, HTR2A and HTR1D; SERT	CRC cells, HCC cells, CD8^+^ T cells, tumour immune niche	HTR2B promotes CRC EMT and metastasis. Serotonin can support HCC growth through mTOR, Wnt/β-catenin, YAP or receptor-related pathways. SERT can restrain CD8^+^ T-cell antitumour immunity by depleting intratumoural serotonin, and SERT inhibition may restore serotonin-dependent immune activity	Distinguish receptor-mediated tumour-cell effects from SERT-mediated immune regulation. SSRIs inhibit SERT rather than blocking 5-HT signalling	Li et al. (2024), Carmi et al. (2025), Peters et al. (2014), Zhu et al. (2022b), Soll et al. (2010)

Within this feedback network, IL-6 is a representative bridge molecule linking the TME to pain plasticity and brain dysfunction. IL-6 can signal through both membrane-bound IL-6R-mediated classical signalling and soluble IL-6R-mediated trans-signalling, thereby activating JAK/STAT 3 and intersecting with inflammatory transcriptional networks such as NF-kappaB to sustain pro-inflammatory and stress-related gene programmes. Notably, the effects of elevated peripheral IL-6 on the brain do not necessarily require overt BBB disruption. Circulating IL-6 can rapidly access the area postrema (AP), which lies outside the BBB, activate AP neurons and associated networks, enhance excitatory synaptic transmission, and induce network hyperactivity; neutralization of IL-6 or selective inhibition of AP neurons can alleviate cachexia-like phenotypes and prolong survival, suggesting that the axis of “peripheral IL-6 - AP -abnormal brain network activity” is a potentially actionable central pathway. At the same time, in the peripheral sensory system, IL-6 can induce MNK1/2-eIF4E-dependent nascent protein synthesis in nociceptors, supporting the notion that cytokine-driven sensory neuroplasticity is an important mechanistic basis for cancer pain and neuropathic pain. This concept is also consistent with findings of TRPV 1 upregulation and functional enhancement in bone cancer pain (Sun et al., 2024; Mitchell et al., 2024; Niiyama et al., 2007). Corresponding to the IL-6 axis, the TNF-alpha axis more prominently reflects inflammation-mediated remodelling of synapses and ion channels (Venkatesh et al., 2019; Venkataramani et al., 2019; Olmos and Lladó, 2014; Sanchez Trivino et al., 2024; Feyissa et al., 2022). Through TNFR1/2, TNF-alpha can rapidly alter the excitatory-inhibitory balance by enhancing AMPA/NMDA-related excitatory transmission while weakening GABA receptor-mediated inhibitory transmission, thereby lowering the neuronal firing threshold and amplifying neuroinflammation and excitotoxicity. This mechanism is particularly relevant to brain tumour-related epilepsy (BTRE). Exosomes derived from human glioma can depolarize the resting membrane potential of hippocampal neurons and induce upregulation of voltage-gated sodium channels via exosomal TNF-alpha; the TNF-alpha blocker infliximab can markedly attenuate this hyperexcitable phenotype. Meanwhile, AMPA receptor-dependent neuron-glioma synapses and electrochemical integration between glioma and host neural networks indicate that seizure-like hyperactivity is not merely a complication of the tumour, but may in turn provide activity-dependent proliferative signals that further support tumour growth and invasion.

Immune cells and extracellular vesicles constitute important amplifiers of this feedback network. In the CNS, systemic inflammation can drive preferential accumulation of microglia around cerebral vessels and regulate BBB integrity, whereas persistent inflammation further promotes expansion of neuroinflammation and synaptic abnormalities (Guo and Gil, 2022). Tumour-derived extracellular vesicles (EVs) can not only act directly on neuronal ion channels and excitability programmes, but also reprogramme macrophage and microglial states, thereby fostering an immunosuppressive microenvironment. Studies on Schwann cells further suggest that tumours can hijack their “repair-like” phenotype to drive perineural invasion, pain sensitization, and immunosuppression (Guo and Gil, 2022; Wang L. et al., 2024; Zhang S. et al., 2024). Thus, mechanistically, EVs, TAMs/microglia, and Schwann cells collectively maintain a more stable inflammatory-neural feedback loop that is more difficult to terminate spontaneously.

Clinically, this framework has two major implications. First, circulating or exosome-associated IL-6/TNF-alpha and their downstream neural phenotypes merit consideration as candidate biomarkers for symptom stratification and treatment monitoring. Second, although inhibitors targeting IL-6/IL-6R and TNF-alpha pathways have accumulated mature experience in inflammatory diseases and have shown exploratory value in settings such as cancer cachexia and neuroinflammatory pain, inflammation itself is a double-edged sword, and excessive suppression may impair antitumour immunity or produce neuroimmune adverse effects. Accordingly, a more rational translational path forward is not broad-spectrum suppression of inflammation, but rather precise anti-inflammatory and neuromodulatory interventions directed at specific cellular sources, specific time windows, and specific neural targets, delivered in synergy with standard anticancer therapies to balance symptom control with antitumour efficacy. Overall, bidirectional regulation between the nervous system and the TME is not dominated by any single molecule, but instead rests on a multilayered network supported jointly by cytokines, neuropeptides, neurotrophic factors, and extracellular vesicles. Downstream, these signals converge mainly on pathways including JAK/STAT 3, MAPK/ERK, PI3K/AKT/mTOR, cAMP/PKA, and NF-kappaB, ultimately driving alterations in neural excitability, immune disequilibrium, and remodelling of the tumour niche.

## 5 Discussion

The core integrative view of this Review is that the bidirectional interactions between the nervous system and the tumour microenvironment can be distilled into a limited set of common feedback loop architectures, rather than a collection of unrelated molecular pathways. This framework is able to unify findings across different tumour types and anatomical sites. To illustrate: when neural signals drive tumour and stromal processes, β-adrenergic, cholinergic, and sensory neuropeptide-related pathways all fall under innervation-driven tumour progression; when the tumour microenvironment actively recruits and remodels neural tissues, processes dependent on nerve growth factors, extracellular vesicles, and Schwann cells are unified as tumour-induced neuralization; regulatory mechanisms involving AMPA receptors, neuroligin-3, the BDNF–TrkB pathway, and tumour microtubes are classified as bidirectional synaptic and electrical coupling; and regulatory pathways mediated by cytokines, lactate, the hypothalamic–pituitary–adrenal axis, and autonomic outputs together constitute systemic neuroimmune feedback. Through this systematic organization, this Review transcends the limitations of previous general overviews and provides researchers with a clear and practical theoretical framework for identifying loop-specific biomarkers and therapeutic targets.

Although cancer neuroscience has rapidly evolved from a descriptive discipline into a mechanism-driven field, its clinical translation still faces substantial challenges. First, the degree of neural dependence differs markedly across cancer types, organs, and even subtypes within the same tumour entity, making many conclusions difficult to extrapolate directly within a pan-cancer framework. Second, most causal evidence still derives primarily from animal models, organoids, and local tissue studies, whereas tumours in patients are far more complex with respect to neural-fibre distribution, receptor-expression profiles, treatment background, and immune status. Thus, the dominant processes and actionable windows corresponding to neural tumour-promoting effects, immune-regulatory patterns, and metabolic associations observed under experimental conditions remain to be defined more clearly in clinical settings. In addition, neural regulation is itself tightly coupled to cardiovascular, gastrointestinal, cognitive, endocrine, and immune homeostasis, and any systemic intervention may therefore be accompanied by non-tumour tissue toxicity and adaptive compensation, representing an unavoidable boundary for clinical application.

A further limitation of the current field is that different types of evidence are often discussed together, although they do not carry the same inferential strength. Causal *in vivo* perturbation studies, such as denervation, receptor knockout or pharmacological blockade, provide stronger mechanistic support than cell-based or organoid experiments. Human pathological findings, such as increased nerve density, perineural invasion or receptor expression, support clinical relevance but are primarily associative. Similarly, epidemiological observations, including associations between beta-blocker use and cancer outcomes, remain vulnerable to confounding and should be considered hypothesis-generating unless supported by prospective mechanistic or interventional studies. Important confounders include baseline psychological stress, cancer-related pain, opioid and non-opioid analgesic use, cardiovascular comorbidities and indications for β-blocker prescription, concomitant medications, dose and receptor selectivity of neural-modulating agents, treatment duration and adherence, and the timing of intervention relative to surgery, chemotherapy, radiotherapy or immunotherapy. Therefore, although we summarize emerging neuro-targeted strategies, many of these mechanisms still require larger-scale preclinical validation, spatially resolved human studies and well-designed prospective clinical trials before their therapeutic relevance in patients can be firmly established. Accordingly, throughout this Review, neuro-targeted interventions should be interpreted as mechanistically informed therapeutic hypotheses at different stages of validation—ranging from preclinical causality and retrospective association to early-phase feasibility, randomized symptom-control evidence and, ultimately, randomized antitumour efficacy—rather than as clinically ready precision-oncology interventions (Tokunaga et al., 2025; Zhang et al., 2025; Fallon et al., 2023). Future studies should further integrate multi-omics approaches with electrophysiological techniques to build an analytical framework in which nerve-fibre distribution, immune-cell states, metabolic gradients, and symptom phenotypes can be resolved within the same domain, thereby identifying targets that are truly pathogenic and pharmacologically actionable. Recent studies in spatial oncology and spatial multi-omics have already shown that such technologies can not only map functional cellular neighbourhoods within the tumour microenvironment, but also link spatial structural features to clinical outcomes, therapeutic responses, and mechanistic stratification. Furthermore, immunotherapy outcome-prediction models built on spatial multi-omics suggest that spatial features may become an important source of next-generation mechanistic biomarkers.

At the same time, future efforts should promote biomarker-based stratification according to potential indicators such as nerve density, receptor expression, neurotransmitter profiles, and neural excitability in order to support mechanism-guided clinical trial design. With regard to treatment strategies, the more promising direction is not simple suppression of all neural input, but rather the development of local, reversible, and monitorable precision neuromodulation approaches tailored to specific cancer types, specific neural pathways, and specific disease stages, in rational combination with immunotherapy, metabolic intervention, and standard anticancer treatment. Recent translational studies in cancer neuroscience have already clearly indicated that tumour-induced neural injury may be associated with poor response to anti-PD-1 therapy (Aung et al., 2025), suggesting that the neural state itself may represent not only a source of symptoms but also an important determinant of therapeutic response.

Overall, the interaction between the nervous system and the tumour microenvironment has progressed from being viewed as a mere accompanying phenomenon of local invasion to becoming an important theoretical framework for explaining remodelling of the tumour niche. The nervous system can shape tumour-cell behaviour and the immunosuppressive microenvironment through neurotransmitters, neuropeptides, neurotrophic factors, and electrophysiological coupling, whereas tumours and their stromal components can in turn induce neuralization, neural sensitization, and neural-network remodelling through inflammatory, metabolic, and extracellular-vesicle-mediated signals. From basic research to clinical translation, cancer neuroscience is driving a paradigm shift in oncology from a cancer-cell-centred perspective towards a systems-network-centred perspective. In the future, with further integration of multimodal omics, functional neuroscience, and precision therapeutic strategies, incorporation of the neural dimension into precision oncology may not only improve antitumour responses but also simultaneously ameliorate pain, epilepsy, cognitive impairment, and other neurologically related outcomes, thereby delivering dual benefits in tumour control and symptom management. Thus, future precision oncology should not only ask whether a tumour is innervated, but also determine which nerve–tumour feedback loop is dominant, which cellular compartment maintains it, and which loop-specific intervention can disrupt it without compromising physiological neural homeostasis.

## Author's note

HJ and YL are the guarantors of this work and take full responsibility for the integrity of the data and the accuracy of the data analysis.
